# Virus ecology of fluvial systems: a blank spot on the map?

**DOI:** 10.1111/brv.12202

**Published:** 2015-06-24

**Authors:** Peter Peduzzi

**Affiliations:** ^1^ Department of Limnology & Bio‐OceanographyUniversity of ViennaAlthanstrasse 14A‐1090ViennaAustria

**Keywords:** stream, river, bacteriophage, organic matter, C‐flux, bacterial–viral loop

## Abstract

The ecology of viruses has been studied only in a limited number of rivers and streams. In light of a recent re‐appraisal of the global fluvial surface area, issues such as abundance and production, host mortality and the influence of suspended particles and biofilms are addressed. Viral life cycles, potential impacts of viruses on water biochemistry and carbon flow, and viral diversity are considered. Variability in trophic levels along with the heterogeneous nature and hydrological dynamics of fluvial environments suggest a prevailingly physical control of virus‐related processes under lotic conditions and more biological control under lentic conditions. Viral lysis likely contributes to a pool of rapidly cycling carbon in environments typically characterized by high proportions of recalcitrant terrestrial carbon. On average, 33.6% (equalling 0.605 Pg C year^−1^) of the globally respired carbon from fluvial systems may pass through a viral loop. Virus distribution and the proportion of organic material in horizontal transport versus processes in retention zones remain to be determined in detail. The need for up‐scaling the contribution of virus‐related processes in fluvial systems is of global relevance. Further, the role of climate change and the effect of anthropogenic alterations of fluvial systems on viruses require attention. The identification of these considerable knowledge gaps should foster future research efforts.

## I. INTRODUCTION

Fluvial waters comprise a small but pivotal fraction of global water resources. They are critical links in global organic matter cycles and budgets. Rivers, their riparian zones and fringing wetlands are hotspots of biodiversity (Junk, Bayley & Sparks, 1989; Newbold, 1992; Battin *et al*., 2009b). Reasonable estimates have been made of global water discharges, but less effort has been devoted to determining the surface area covered by the global river network (Downing *et al*., 2012). Based on novel approaches to approximate the size distribution and areal extent of streams and rivers, new estimates of the global fluvial area are 30–300% greater than previously published appraisals (Downing *et al*., 2012). This supports the current perception that the contribution of river networks to fluvial net heterotrophy and CO_2_ outgassing was underestimated in the past (Battin *et al*., 2008; Raymond *et al*., 2013). Moreover, humans have extensively altered river systems by fragmentation and flow regulation, triggering various ecological shifts and risks (Nilsson *et al*., 2005).

Viruses are the smallest and most numerous biological entities in aquatic ecosystems, with the majority being bacteriophages (Wommack & Colwell, 2000; Weinbauer, 2004; Suttle, 2007). Abundances of planktonic viruses commonly range between 10^4^ and 10^8^ ml^−1^ and are generally higher in inland waters than in marine systems. Peak values have been reported for very productive estuaries and lakes (Peduzzi & Luef, 2009). Exceptionally high virioplankton abundance (up to 2 × 10^9^ ml^−1^) has been documented in the alkaline, hypersaline Mono Lake, California (Brum *et al*., 2005), and very recently in the alkaline–saline Lake Nakuru in Kenya, East Africa (up to 7 × 10^9^ ml^−1^; Peduzzi *et al*., 2014). The typically high frequency of virus infection in oceanic ecosystems is known to be a major cause of prokaryotic host mortality. Host cell lysis in the marine environment was calculated to be a source of up to 10^9^ tons of carbon per day released from the biological pool (Suttle, 2007; Brussaard *et al*., 2008). The biogeochemical consequence is altered rates of carbon accumulation in the photic zone (CO_2_ release to the atmosphere *versus* vertical transport to the meso‐/bathypelagic zone). Viruses are also involved in shaping the composition of bacterial communities, either by reducing abundant host taxa or by introducing new genetic information into their hosts. They may be the largest genetic reservoir on earth and may boost the resilience of ecosystems by sustaining multiple species with similar or identical biochemical pathways (compare Thingstad, 2000; Brussaard *et al*., 2008; Jacquet *et al*., 2010). We need a better understanding of the role of viruses, including the use of new approaches in future studies. Whole‐genome shotgun metagenomics, single virus genomics or targeted viromics, with next‐generation sequencing, will likely be standard in future diversity research (Thurber *et al*., 2009; Zeigler Allen *et al*., 2011; Adriaenssens & Cowan, 2014; Martínez Martínez, Swan & Wilson, 2014). Further, research on archaeal viruses, their diversity and potential ecological significance is at an early stage. Most isolates stem from geothermally heated environments and from halophilic archaeal hosts (Prangishvili, Forterre & Garrett, 2006). Their role in fluvial systems remains unexplored.

Surprisingly, for running water systems, many aspects of aquatic virus ecology are underrepresented or ignored. Recently, however, interest among limnologists and microbial ecologists is increasing (Peduzzi & Luef, 2009; Jacquet *et al*., 2010; Pollard & Ducklow, 2011; Peduzzi, 2013). This review covers naturally occurring viruses infecting microbial hosts present in lotic systems, and does not consider the distribution and survival of animal and human pathogenic viruses. The importance of non‐endemic hosts is evident and has been extensively reviewed previously (e.g. Goyal, Gerba & Britton, 1987). The current review paper provides: (*i*) an overview of existing information on virus ecology in fluvial waters by tackling issues such as virus‐mediated microbial host mortality, viral lifestyles and mechanisms controlling viral activity; (*ii*) an assessment of the impact of variable hydrology and the role of suspended particles and biofilms on viruses; (*iii*) information on the need to investigate horizontal transport processes of virus‐related solutes and particles, and virus dissemination; and (*iv*) arguments for the necessity of up‐scaling the contribution of virus‐related processes, for example their potential contribution to global CO_2_ outgassing and the role of climate change on these contributions. These considerations include the significance of anthropogenic alterations of fluvial systems and, together, will help define research fields for future studies.

## II. VIRAL ABUNDANCE, DISTRIBUTION AND DYNAMICS IN FLUVIAL SYSTEMS

Little is known on how the fundamental (abiotic and biotic) differences between marine, lacustrine and running waters affect the abundance, composition, production and control mechanisms of viruses. Generally, as a result of the balance between production and decay, virus numbers are linked to system productivity. Eutrophic water bodies typically contain more virus particles than oligotrophic systems (see Jacquet *et al*., 2010, and references therein). Nonetheless, productivity, trophic state and the abundance of viruses can change seasonally, particularly in running waters with pronounced hydrological dynamics. Based on an earlier compilation of inland waters (Peduzzi & Luef, 2009, and references therein) and including more recent data, virus abundances in the water column of different fluvial systems range from 0.007 to 88.8 × 10^7^ ml^−1^ (Table 1); extremely low values such as 0.009 × 10^7^ ml^−1^ from the Talladega wetland (Farnell‐Jackson & Ward, 2003) may also reflect methodological constraints in this and older studies. Nonetheless, the variability in flowing waters is large and clearly driven by dynamic and variable hydrology. The high degree of spatial heterogeneity in such systems (e.g. backwaters with often lacustrine conditions) also plays a role (Peduzzi & Luef, 2008, 2009; Jacquet *et al*., 2010; Ma *et al*., 2013). Finally, virus abundance apparently varies more on seasonal scales in inland waters than in the marine environment (Wilhelm & Matteson, 2008).

**Table 1:** Viral abundance, virus‐prokaryote ratio (VPR), burst size, frequency of infected cells (FIC), frequency of visible infected cells (FVIC), fraction of mortality due to viral lysis (FMVL), viral production and proportion of lysogenic bacteria in various fluvial waters; data are given as range (average)

Location	Viral abundance (× 10^7^ ml^−1^)	VPR	Burst size	FIC (%)	FVIC (%)	FMVL (% of the total bacterial population)	Viral production (× 10^8^ VLPs l^−1^ h^−1^)	Lysogenic bacteria (% of the total bacterial population)	Reference
**Running waters**									
Ria de Aveiro (Portugal)	2.4–25 (TEM)	4.7–55.6 (18)				49–74			Almeida *et al*. (2001)
Bremer River (Australia)	1.2–13.5 (EFM)	3.04–35.16				0–100	1702		Pollard & Ducklow (2011)
Brisbane River/Moreton Bay, Noosa River (Australia)	0.5–30 (EFM)	3–37					<0.01–23		Hewson *et al*. (2001)
Charente River (France)	3.9–9.8^a^ (EFM)	11.3–14.3^a^							Auguet *et al*. (2005)
Danube River (Austria)	0.32–3.5 (2.09) (EFM)	4.11–35.0 (19.8)							Peduzzi & Luef (2008)
Danube River (Austria)	1.9–7.23 (EFM)	5.7–27.5							Besemer *et al*. (2009)
Ditch (Port Aransas, USA)	14.6 (TEM)								Hennes & Suttle (1995)
Djeuss Stream (Senegal)	1.1 (EFM), 2.6 (TEM)	4.1	34	2.1^b^	0.3	2.5	4.10	7.1	Bettarel *et al*. (2006)
Ephemeral channel (Charles City, USA)	1.0–7.0 (EFM)								Williamson *et al*. (2014)
Haihe River (China)	7.35–88.8 (FCM)	5.12–46.9 (21.4)							Ma *et al*. (2013)
Mahoning River (USA)	0.2–2.02 (EFM)	0.42–12.2							Lemke *et al*. (1997) and Baker & Leff (2004)
Morava River (Czech Republic)	0.17–4.74 (EFM)								Slováckova & Marsálek (2008)
Nile River (Cairo, Egypt)	7.75 (EFM)	14.24							Peduzzi *et al*. (2013)
Svratka River (Czech Republic)	0.19–5.81 (EFM)								Slováckova & Marsálek (2008)
Wadi Hatta (UAE)	0.89 (EFM)	6.61							Peduzzi *et al*. (2013)
Yangtze river estuary (China)	0.07–1.68 (FCM)	1.52–72.0 (8.7)							Jiao *et al*. (2006)
**River reservoirs**									
Reservoirs (Sri Lanka)	3.1–7.7 (EFM)	9.7–23.8		10.8–26.9	1.6–4.4	13.2–46.1			Peduzzi & Schiemer (2004)
Rímov Reservoir (Czech Republic)	1.6 (EFM)		19						Simek *et al*. (2001)
Grim Dell Pond (Charles City, USA)	0.36–1.35 (EFM)								Williamson *et al*. (2014)
**Floodplains, wetlands**									
Alte Donau (Austria)	1.7–11.7 (5.0) (TEM)	4–39 (19)	18–48	10–63 (28)	2.8–9 (5)	26–125			Fischer & Velimirov (2002)
Amazon floodplain lake (Brazil)	0.5–1.7 (EFM, TEM)	4.4–6.0^a^	10		20				Barros *et al*. (2010)
Floodplain, Danube River (Austria)	0.31–10.2 (2.62) (EFM)	2.91–33.9 (13.3)							Luef *et al*. (2007)
Floodplain, Danube River (Austria)	1.78–15.3 (EFM)	2.5–15.0							Besemer *et al*. (2009)
Danube River floodplain segments								5.83–25.0	Tvarogova (2010)
Kühwörther Wasser, Danube River backwater (Austria)	1.2–6.1 (TEM)	2.0–17.0	15.5–38.0	5.4–21.6		10.8–43.2			Mathias *et al*. (1995)
Lobau, Danube River backwater (Austria)	2.4–10.6 (6.38) (EFM)	14.0–48.2 (7.23)							Peduzzi & Luef (2008)
Talladega Wetland (USA)	0.009–0.12 (EFM)	0.02–2.46							Farnell‐Jackson & Ward (2003)
Trombetas River (Brazil)	0.4–3.0 (EFM)	2.2–9.1							Almeida *et al*. (2015)
**Others**									
Barton Spring (Texas, USA)	0.53 (EFM), 0.39 (TEM)								Hennes & Suttle (1995)
Hot springs (California, USA)	0.007–0.7 (EFM)						10.0–15.0		Breitbart *et al*. (2004)
Marsh	57.4–70.0 (EFM), 22.7 (TEM)								Hennes & Suttle (1995)
**Benthic, running waters**									
Brisbane River/Moreton Bay, Noosa River (Australia)	0.2–4.8 × 10^9^ cm^−3^ sediment (EFM)	2–65							Hewson *et al*. (2001)
Djeuss Stream (Senegal)	15.5 × 10^7^ ml^−1^ (EFM), 8.5 × 10^7^ ml^−1^ (TEM)	1.3			<0.1			1.9	Bettarel *et al*. (2006)
Esino River (Italy)	9.83 × 10^8^ ml^−1^ (sediment) (EFM)	0.77	16.6			18.4	770.0	1.48	Mei & Danovaro (2004)
Mahoning River (USA)	1.65–6.68 × 10^8^ g^−1^ AFDM in particulate samples (EFM)	0.2–1.2							Lemke *et al*. (1997)
Mahoning River (USA)	4.71–8.91 × 10^6^ g^−1^ sediment (EFM)	Sediment: 0.09–0.45							Baker & Leff (2004)
	4.81–21.8 × 10^8^ g^−1^ leaf (EFM)	Leaf: 0.40–2.55							

AFDM, ash‐free dry mass; EFM, epifluorescence microscopy; FCM, flow cytometry; FIC, frequency of infected cells; FMVL, fractional mortality due to viral lysis; FVIC, frequency of visibly infected cells; TEM, transmission electron microscopy.

a Range of means.

b Calculated, using the formula: FIC = 7.11 × FVIC.

Several methods are available to enumerate virus particles. In virus ecology, culture‐based methods are inefficient for quantifying natural virus abundances (Weinbauer, 2004; Peduzzi & Luef, 2009). At present, the most widely used methods for water samples are based on direct counts using either transmission electron microscopy (TEM), epifluorescence microscopy (EFM) or flow cytometry (FCM). Typically, viral abundance exceeds that of bacteria, which can be defined by a virus/prokaryote ratio (VPR) as a descriptor of the relationship between viruses and potential hosts. For inland waters (including lakes), the VPR typically ranges between 1 and 100, with an overall mean of around 20–25, and is more variable than in marine waters (Wilhelm & Matteson, 2008; Peduzzi & Luef, 2009). VPR values tend to be higher for more nutrient‐rich, productive environments, thus often being higher in limnetic compared to marine systems (see reviews by Weinbauer, 2004; Peduzzi & Luef, 2009; Jacquet *et al*., 2010). In some Antarctic lakes, for example, VPR values up to 150 have been reported (Kepner, Wharton & Suttle, 1998; Madan, Marshall & Laybourn‐Parry, 2005). In marine pelagic waters the VPR typically ranges from 5 to 15 (Weinbauer, 2004; Suttle, 2007), but exceptions occur (e.g. some deep‐sea values exceed 100; Suttle, 2007).

From both marine and lake environments we know that microbial (host) activity supports high viral production. In running water systems, information on viral production rates is conspicuously scarce, but the rates seem to vary over several orders of magnitude (Table 1). Examples include very low values occasionally found in some Australian rivers and the Djeuss stream, Senegal (Hewson *et al*., 2001; Bettarel *et al*., 2006) to very high values of 170.2 × 10^9^ l^−1^ h^−1^ in another Australian eutrophic river (Bremer River; Pollard & Ducklow, 2011). Productivity in the sediments of the Esino River (Italy) was 77.0 × 10^9^ viruses l^−1^ h^−1^ (Mei & Danovaro, 2004).

## III. VIRAL HOST MORTALITY

As in the marine environment, in inland aquatic systems viral infection of both auto‐ and heterotrophic bacteria and subsequent lysis of host cells is responsible for a considerable proportion of prokaryotic mortality (Peduzzi & Luef, 2009). Virus‐induced mortality of prokaryotes is often expressed as the fraction (%) of mortality due to viral lysis (FMVL). Even though the various methods for assessing this are subject to bias from many sources, in aquatic systems (both marine and freshwater) typically 10–20% of bacterial production is lost due to virus infection (Jacquet *et al*., 2010). When prokaryotes, such as cyanobacteria, are important members of the food chain, their lysis can have adverse effects even for vertebrate end‐consumers (Peduzzi *et al*., 2014).

In the water and sediment of fluvial systems, values between 2.5% and above 70% have been reported; at times virus infection seems to be responsible for the entire mortality of their hosts (Table 1). In an experimental approach, Ortmann *et al*. (2011) found that viral lysis varied for different components (auto‐ and heterotrophic) of the microbial community across an estuarine gradient (Mobile Bay, USA). In the silty sediment layer of a floodplain lake between 0 and 36% of bacterial secondary production was removed by viruses, and the viral impact prevailed over protozoan grazing by a factor of 2.5–19.9 (Fischer *et al*., 2003, 2006). A recent study estimated bacterial mortality in the subtropical Bremer River (Australia) to average 3.40 × 10^9^ bacterial cells l^−1^ h^−1^ (Pollard & Ducklow, 2011). Again this viral parameter is also likely to be quite variable. Using the available data from fluvial systems we calculated a mean ± S.D. FMVL of 33.6 ± 28.1%. Thus, besides grazing by protists, viruses are clearly an important factor exerting control on their host cells in fluvial systems. Viruses and flagellates probably act additively in their controlling effects on prokaryotes. More information on virus‐ *versus* grazer‐related mortality is available for freshwater than marine environments (Wilhelm & Matteson, 2008). In some anoxic lakes, solar salterns and polar lakes (i.e. inland lakes), virus‐related prokaryote mortalities are among the highest (up to 100%) reported so far, matching or even exceeding grazer‐mediated mortality. Also burst size (i.e. the number of virus particles released from a cell upon host lysis) appears to be on average larger in freshwater (10–50) than in marine environments (20–25; Wilhelm & Matteson, 2008 and references therein). Higher values have been reported in phytoplankton (cyanobacteria and eukaryotic algae), often ranging from 100 to >500 (sometimes to a few thousand), probably linked to the often greater host cell volume (Wilhelm & Matteson, 2008; Jacquet *et al*., 2010). The high variability in burst size is presumably linked more strongly to the trophic status of a system than to its salinity (Parada, Herndl & Weinbauer, 2006). For marine environments, the factors affecting virus dynamics and microbial host–virus interactions were reviewed recently (Mojica & Brussaard, 2014). For fluvial systems, no synopsis exists.

Viruses that infect eukaryotic algae have been described in detail by Van Etten, Lane & Meints (1991). In particular *Chlorella* viruses were detected in freshwater samples across Japan and in several countries of Asia, Europe, North America and South America, and investigated in detail (Yamada *et al*., 1993). The Phycodnaviridae family is described as important in infecting and causing mortality of eukaryotic phytoplankton all over the world (Suttle, 2007). Genome sequences of 41 chloroviruses from terrestrial water samples throughout the world were recently analysed in detail (Jeanniard *et al*., 2013). Furthermore, sequences homologous to the chlorovirus *Acanthocystis turfacea* chlorella virus 1 (ATCV‐1) were unexpectedly extracted from human oropharyngeal samples; this virus caused harm to the human host (Yolken *et al*., 2014). For fluvial systems, however, almost no information exists on eukaryotic phytoplankton viruses.

There is still no standard approach for estimating and comparing mortality in prokaryotic and eukaryotic communities that can be attributed to viruses in different aquatic environments. This prevents the accurate integration of virus‐related mortality into overall models (Jacquet *et al*., 2010), particularly for riverine systems.

## IV. PARTICLE‐ AND BIOFILM‐ASSOCIATED VIRUSES

Flowing waters are often particle‐rich environments. Beyond the typically pronounced environmental heterogeneity on the fluvial landscape scale, this creates microscale patchiness for microorganisms. Reliable data are even scarcer; one example is from the Danube River, with up to 5.39 × 10^9^ viruses cm^−3^ suspended river particle volume (Luef, Neu & Peduzzi, 2009a). Microscale patchiness has been studied in detail in marine and estuarine habitats (Seymour *et al*., 2006; Azam & Malfatti, 2007; Stocker, 2012). The highest particle‐associated viral abundance in a natural aquatic system so far (up to 30 × 10^10^ viruses ml^−1^) was found on suspended aggregates in a semi‐enclosed bay in the southwest lagoon of New Caledonia influenced by terrestrial input (Mari, Kerros & Weinbauer, 2007). It was estimated that up to 35% of the viruses in Danube River water were associated with suspended matter (Luef *et al*., 2007, 2009a).

Beyond particle quantity, quality also seems to be pivotal for viral abundance in fluvial systems (Luef *et al*., 2007; Peduzzi & Luef, 2008; Kernegger, Zweimüller & Peduzzi, 2009). In a river‐floodplain system of the Danube, the quality, size and age of particles and aggregates, and exposure time of viruses to aggregates, were key factors regulating viral abundance (Kernegger *et al*., 2009). Importantly, particles (mainly with organic constituents) apparently play a role as viral scavengers by removing viruses through adsorption, or as reservoirs rather than viral factories (Weinbauer *et al*., 2009). The presence/abundance of suspended particles appears to influence the overall (free and attached) virus distribution. That is, as particles with organic constituents increased, more viruses were attached and fewer were freely suspended (Peduzzi & Luef, 2008). Such particles apparently remove viruses from the water column, with potentially considerable consequences for various aspects of river ecosystems and related biogeochemical fluxes. Moreover, viral lysis can influence the formation, size and stability of suspended aggregates (Peduzzi & Weinbauer, 1993; Weinbauer *et al*., 2009). Thus, in flowing waters at least two aspects are pivotal: the consequences for horizontal transport of material, and the role of particles in the distribution and spreading of viruses along the flow continuum and finally to the sea. More comprehensive studies have been hampered by the complicated techniques required to make reasonable estimates of the number of particle‐associated viruses (see Peduzzi & Luef, 2009; Luef *et al*., 2009a,b; Peduzzi, Agis & Luef, 2013). It remains unclear to what extent viruses are truly attached or distributed in the pore water of suspended matter and aggregates (Weinbauer *et al*., 2009).

The benthic zone and river‐bank are especially important habitats for viruses in rivers. The available information on viriobenthos in both freshwater and marine sediments was reviewed earlier (Danovaro *et al*., 2008a; Pinto, Larsen & Casper, 2013). These meta‐analyses highlighted a large variability of viral abundance with values ranging between 10^4^ and 10^11^ viral particles ml^−1^ of wet sediment. Comparing this broad range with values from suspended particles implies some commonalities but also variability. One reason for the potential differences is the drastic change in conditions (anoxia) in deeper layers of the benthos (see Pinto *et al*., 2013).

Viruses also exist at the surface layer of the benthos. An experimental study on wetland biofilms revealed that virus‐sized microspheres can be embedded to a 100‐fold increase above the maximum water column abundance and remain at >1.92 × 10^9^ ml^−1^ for several months (Flood & Ashbolt, 2000). Recent studies on the persistence of specific pathogenic viruses in river water imply that water temperature is important. Riverbank filtration is potentially an efficient process in removing contaminants such as virus particles from infiltrated surface water. On the one hand, temperature influences virus decay rates, while on the other hand the changing viscosity and density effects of water during riverbank filtration can counteract with temperature‐dependent decay and inactivation rates (Derx *et al*., 2013; Yang & Griffiths, 2013). This might be relevant in the future because along the Austrian stretch of the Danube for example, mean water temperature has increased (long‐term trend since 1951) and will likely continue to do so (Zweimüller, Zessner & Hein, 2008).

A general gap in our knowledge about sediment‐ and particle‐associated viruses (particularly for fluvial systems) is evident and should be addressed in future work.

## V. VIRAL PROLIFERATION CYCLES

The two most important types of viral life cycles are represented by lytic and lysogenic infections. Highly variable environmental conditions probably favour one or the other of these pathways, especially in fluvial waters. Their relative importance is crucial for the host community and for many aspects of the microbial loop (see e.g. Peduzzi & Luef, 2009). During viral lysis, new virus progeny are released from the host cell; whereas in the lysogenic cycle, the genome of a ‘temperate’ virus remains dormant within the host cell until a lytic cycle is induced. If lysogeny prevails and the main control of prokaryotic abundance is *via* protozoan grazing, most of the carbon will be channelled to higher trophic levels (the microbial loop). By contrast, if viral lysis accounts for most prokaryotic losses, then carbon and nutrients are diverted away from larger organisms (Proctor & Fuhrman, 1990; Wilhelm & Suttle, 1999).

Unfortunately, for fluvial systems few publications are available on the proportion of lytic *versus* temperate viruses. The proportion of lysogenic cells *versus* lytic cells in the Djeuss stream (Senegal, Africa) was variable, averaging 7.1%; in reservoirs fed by the Senegal River the percentage was much lower (0.1–2.5%; Bettarel *et al*., 2006). Preliminary data from a survey conducted in a river‐floodplain section of the Danube (Austria) revealed a low frequency of lysogenic cells in a frequently isolated, productive floodplain section (average 5.83%). The mean value was higher in a frequently (>200 days year^−1^) flushed section (25.0%) and in the main channel (21.2%) (Tvarogova, 2010). The lytic–lysogenic state may be influenced either by the interplay between demand and availability of host‐limiting nutrients due to increased cellular requirements during virus replication, or even be understood as a type of ‘survival’ strategy for viruses under different environmental conditions (Weinbauer, 2004; Ankrah *et al*., 2014). Based on studies in both marine and lake environments, changes in system productivity are an important factor. It is believed that lysogeny enables viruses to survive within host cells under oligotrophic conditions, and lytic infection leads to rapid propagation when host abundance and productivity are high (Weinbauer *et al*., 2003; Payet & Suttle, 2013; Pradeep Ram *et al*., 2014). In the Bach Dang Estuary (Vietnam), the salinity gradient from fresh to marine water induced a spatial succession of the two viral life strategies (Bettarel *et al*., 2011). A recent study conducted in coastal lagoons with highly variable conditions provides a more detailed view on regulating factors (Maurice *et al*., 2013). They reported that the lytic cycle was triggered by environmental parameters and host physiology, whereas lysogeny seemed to depend on the variability of prokaryote physiology. Lysogeny, however, was not affected directly by the level of host activity, responding rather to the amplitude of change in key features of host physiology. It remains to be investigated whether this physiological variability is either a signal to enter the lysogenic replication cycle or to remain in the lysogenic state (Maurice *et al*., 2013). These findings support the view that the two viral lifestyles are dynamic processes that respond on short temporal and spatial scales. This is particularly true in transition systems, where highly fluctuating environmental conditions can modulate host physiology rapidly and significantly. Thus, in fluvial systems, the variable hydrology probably greatly influences the lytic–lysogenic state. Dynamic hydrological situations (flow conditions, flooding) may dilute host abundance and create unfavourable conditions for host production and lytic propagation, thus favouring lysogeny. High percentages of lysogenic cells were also recorded in Lake Bourget (France) when bacterial (host) abundance and activity were lowest (Thomas *et al*., 2011). By contrast, in river systems after a flood, which typically introduces nutrients, host productivity is boosted and may thus promote the lytic cycle (Peduzzi & Schiemer, 2004; Peduzzi & Luef, 2008). This view is mirrored by the above‐mentioned lower frequency of lysogenic cells in the more lentic, productive conditions of the Danube river system *versus* higher values under more turbulent, flowing conditions with diluted host abundance and lowered productivity. This interpretation is in line with the previously reported conclusion that, in fluvial systems, lentic conditions promote prevailingly biological control on the virus–host interaction whereas under lotic conditions physical factors such as gauge, water current, dilution and temperature are more important (Peduzzi & Luef, 2008).

## VI. VIRAL IMPACT ON WATER BIOCHEMISTRY AND CARBON FLOW

In planktonic food webs viral lysis efficiently directs carbon away from biomass into the dissolved organic carbon (DOC) pool at the expense of bacterio‐ and phytoplankton grazing (‘viral shunt’; Wilhelm & Suttle, 1999). A varying but substantial fraction of this organic material can be more or less efficiently utilized by uninfected heterotrophic prokaryotes, thus re‐entering the food web. The net effect of the so‐called ‘viral loop’ is that it ultimately converts organic matter into dissolved inorganic nutrients, including respired CO_2_ (Wilhelm & Suttle, 1999; Haaber & Middelboe, 2009; Pollard & Ducklow, 2011). Studies with marine microorganisms have revealed that viruses can be key players in nitrogen cycling (Haaber & Middelboe, 2009; Shelford *et al*., 2014). In culture experiments, phage infection alters both host metabolism and lysate composition (Weinbauer & Peduzzi, 1995; Ankrah *et al*., 2014; Sheik *et al*., 2014). In the river‐influenced False Creek (Vancouver, Canada), experiments demonstrated that viral lysis leads to ammonium production from liberation of dissolved organic N that is re‐mineralised by uninfected bacteria, thus fuelling primary production (Shelford *et al*., 2012). Nonetheless, a net uptake of ammonium by non‐infected bacteria in other culture experiments revealed that the metabolism of viral lysates can also result in net consumption of inorganic N (Shelford *et al*., 2014). This recent evidence suggests that viral lysis of microbes changes the relative distribution of dissolved organic (and also inorganic) matter with many indirect effects on the aquatic systems (Weitz & Wilhelm, 2012). A recently published multitrophic model on the effects of marine viruses concluded that ecosystems with high densities of viruses will have increased organic matter recycling, reduced transfer to higher trophic levels and increased net primary production (Weitz *et al*., 2014).

Most fluvial systems harbour and transport high amounts of organic carbon (dissolved and particulate). Even in cases where viral lysis releases only a small fraction to the large fluvial organic carbon pool, it could still contribute significantly to the readily utilizable carbon (labile and semi‐labile fraction). This is particularly relevant in these systems, which are typically characterized by a high proportion of allochthonous (terrestrial) aged and recalcitrant carbon. In Danube floodplain areas, hydrological events substantially influence dissolved organic matter (DOM) composition in backwaters, and DOM quality is an important factor regulating bacterial production (Peduzzi *et al*., 2008; Sieczko & Peduzzi, 2014; Sieczko, Maschek & Peduzzi, 2015). Pollard & Ducklow (2011) reported for the Australian Bremer River that terrestrial DOC was partly returned to the atmosphere as CO_2_ through bacterial respiration, assisted by bacteriophage lysis of their hosts. This short‐circuits the microbial loop. For fluvial environments global net heterotrophy is known to be high, stemming largely from microbial respiration (Battin *et al*., 2008, 2009a). In a meta‐analysis from literature data, and by averaging fractional host mortality due to viral lysis (FMVL) in fluvial systems and wetlands (see Table 1), a first estimate is that a mean ± S.D. proportion of 33.6 ± 28.1% (*N* = 22) may pass the viral loop. Combining this with a global appraisal of CO_2_ evasion data for streams and rivers (Raymond *et al*., 2013), it would represent a contribution of 0.605 ± 0.507 Pg C year^−1^ (*N* = 22) to the globally respired carbon. For comparison, in the oceanic environment the viral shunt was estimated to release 0.37–0.63 Pg C year^−1^ on a global scale (Wilhelm & Suttle, 1999; Danovaro *et al*., 2008b). The estimate for fluvial systems presented here appears to be high (compared to the large oceanic water surface area), however, inland waters are where the highest concentrations of DOC occur, with high amounts of terrestrial carbon (Raymond *et al*., 2013; Amado *et al*., 2015). They are generally supersaturated with CO_2_, and particularly streams and rivers are understood as hotspots for gas exchange (Raymond *et al*., 2013). Photochemical degradation is not considered here, since the share of photochemical and biological processing of DOC remains too poorly understood to achieve accurate predictions so far (Amado *et al*., 2015). Nonetheless, by this process, a substantial fraction of carbon may bypass the bacteria–virus loop. Further, our estimated FMVL was restricted to available values from water column processes of riverine environments, since the virus‐related mortality in porewater and biofilms is largely unknown (see Section IV.). Benthic FMVL‐values might be much lower, potentially making our global appraisal an overestimate. Nonetheless, a huge amount of both autochthonous and terrestrial carbon is in transport in running waters, and even if the virus lysis‐related C‐flux in fluvial systems might be very variable, this process is probably significant on a global scale. However, there is still a large amount of uncertainty associated with these estimates, calling for enhanced research efforts. Comprehensive studies on the significance of the viral shunt in aquatic systems are still scarce, and additional data are needed, particularly for flowing inland waters. A recent modelling approach that quantified the effects of marine viruses suggested that all efforts to predict carbon and nutrient cycling without considering virus‐related processes are likely to miss essential features of food webs that regulate global biogeochemical cycles (Weitz *et al*., 2014).

At present it remains largely unclear how climate change will impact viral processes and related element cycles. Nonetheless, there is some degree of consensus that the effect of global temperature increase will be significant (Danovaro *et al*., 2010; Peduzzi, in press). For example viruses have the potential to interact with climate through their contribution to the DOM‐pool and to biogenic particles. In fluvial waters, climate change‐related temperature shifts may well influence the role of viruses by determining the proportion of organic material in horizontal transport and in processing and re‐mineralisation (Peduzzi, in press). In this context it can be concluded that viruses in fluvial systems will be significantly influenced by climate change. Moreover, viruses, in turn, can influence those processes contributing to climate change.

## VII. VIRUS AND HOST DIVERSITY

Based on the presumed species‐specificity and density‐dependence of infection, viruses are thought to play a substantial role in regulating prokaryotic community composition (Thingstad & Lignell, 1997; Fuhrman & Schwalbach, 2003). There are surprisingly few observational or experimental demonstrations of this effect; almost nothing is available for fluvial waters. Incubation experiments with marine microbial assemblages demonstrated that viruses may significantly influence bacterioplankton community structure. This influence, however, was not consistent between sampling locations or different water masses (Hewson & Fuhrman, 2006). Other experiments investigating nutrient‐manipulated marine mesocosms revealed an important link between the bacterial and viral communities (Sandaa *et al*., 2009). Experiments with lake water, combining the effect of viruses and small grazers on the bacterial community, demonstrated that both act as structuring factors (Jacquet *et al*., 2013; Pradeep Ram & Sime‐Ngando, 2014). Their effects, however, may differ, with viruses for example sustaining and grazers reducing bacterial community composition (Jacquet *et al*., 2013). In a Danube River–floodplain system, the bacterial community structures of the river and the various floodplain pools, as well as those of particle‐associated and free‐living bacteria, differed significantly (Besemer *et al*., 2005). Community shifts in prokaryote hosts are probably strongly linked with changes in viral diversity. In a first screening of the virus community structure in the above floodplain system, we found that different habitats (main channel, dynamically connected and isolated sites) contained distinct viral communities with characteristic dynamics over time (Peduzzi, 2013). The community profile at the dynamically connected site was similar to that of the main river channel when connected, but diverged over time when the site was disconnected at low gauge during 4 months. The permanently isolated section exhibited a different development with a more stable virus community profile and larger genome sizes (based on pulsed field gel electrophoresis). The apparent richness was higher in the two floodplain sections compared to the main stem. Only in the permanently isolated section did the apparent richness correlate significantly with bacterial secondary production, indicating a link with host activity under lentic conditions (Peduzzi, 2013). Hydrology seems to be important, as storm events also drastically changed the viral community during a field study in an ephemeral channel and a stormwater retention pond in Virginia, USA (Williamson *et al*., 2014).

## VIII. DISCUSSION AND FUTURE DIRECTIONS

Viruses infect nearly all forms of cellular life, including bacteria, archaea and microeukaryotes. Our growing knowledge on virus ecology of marine and lake environments has increased the incorporation of virus‐related processes into aquatic food‐web models (Weitz *et al*., 2014, and references therein). In these environments, viruses commonly represent about 5% of the carbon fixed in bacteria, and the viral biomass is around 25‐fold larger than the carbon in protists (see e.g. Peduzzi & Luef, 2009). One may expect a similar contribution of viruses in fluvial systems, albeit more variable due to variable hydrology. More important than carbon biomass, however, is virus bio‐reactivity. In the fluvial realm, virus‐related mechanisms appear to be principally similar to other aquatic environments. Nonetheless, the magnitude and effectiveness of certain processes are different, and much more strongly triggered by the dynamic and heterogeneous nature of these systems.

**Figure 1:**
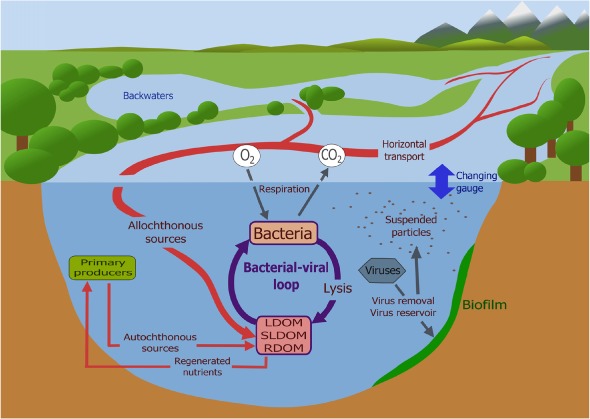
Schematic outline of selected conditions, features and cycles that either are influenced by viruses or influence virus‐related processes in flowing water systems. Based on a synthesis from the existing literature, the most important regulating factors are variable hydrology and environmental heterogeneity; suspended particles and biofilms can remove viruses from the water column and/or act as virus reservoirs; LDOM, labile dissolved organic matter; RDOM, refractory dissolved organic matter; SLDOM, semi‐labile dissolved organic matter. Image credit: Katalin Demeter.

As a synthesis of available information from the literature, Table 2 and Fig. 1 provide an overview of selected conditions and features that influence virus‐related processes in fluvial systems. Perhaps the most important factor regulating these processes is the variable hydrology of running waters. It can either create very unfavourable conditions (temperature, currents, turbulence) for microbial life, or boost production *via* transported nutrient supply. Nothing is known about whether disturbances by flooding influence viral‐induced mortality of prokaryotes. Importantly, variable hydrology also creates edaphic heterogeneity, particularly in more pristine fluvial systems (flowing channels, backwaters, retention zones, lentic water bodies in floodplains). This often leads to a continuum of trophic levels, from oligo‐ to eutrophic subsystems on narrow spatial and temporal scales (Peduzzi & Luef, 2008; Jacquet *et al*., 2010). More open and strongly hydrologically controlled areas can be distinguished from backwaters and retention zones, the latter expanding the opportunity for biological processes.

**Table 2:** Selected conditions and processes influencing the ecological significance of the bacterial–viral loop in fluvial systems. Most factors are controlled by a continuum between physical and biological forces, with one or the other usually prevailing; important factors and environmental settings to be considered are listed

	Prevailing type of control	Factors to be considered
**Influenced by**		
Hydrology	Physical	Proportion of allochthonous/autochthonous material, general environmental setting
Unfavourable conditions	Physical	Temperature, hydrodynamics, mixing, stagnation
Trophic level	Biological	Productivity, nutrients, contact and virus propagation rates
Removal by particles/biofilms	Physical/biological	Current flow and turbulence, particle load and quality
**Effect on**		
Local processing of OM	Biological	Availability of backwaters, retention zones, lentic water bodies
Horizontal transport (export) of OM	Physical	Flow regime, stream and river regulation, loss of habitat heterogeneity
Respiration, CO_2_ outgassing	Physical/biological	Surface area, floods, gas transfer velocity
Viral (host) diversity	Biological	Habitat heterogeneity, viral infectivity and lifestyles

OM, organic matter.

Suspended particles are apparently important in fluvial systems. Complex floating aggregates can develop in larger rivers, reservoirs, floodplain lakes and drowned estuaries, whereas attached biofilms tend to develop in headwaters and streams, fostered by the water current (Battin *et al*., 2008). These structures in fluvial systems apparently influence virus‐related processes differently and potentially more severely than in lacustrine and oceanic environments. Particles and aggregates appear to play the role of viral scavengers or reservoirs rather than viral factories (Weinbauer *et al*., 2009). Adsorption to suspended solids can even stimulate growth of the free‐living prokaryotes, for example by reducing viral lysis. This may also affect microbial diversity, food‐web structure and biogeochemical cycles (Peduzzi & Luef, 2008; Weinbauer *et al*., 2009; Luef *et al*., 2009a,b). In the future improved knowledge on viral and related host diversity should include larger metagenomic studies in fluvial systems, with functional genomics and proteomics hopefully helping to unravel many of the important questions.

In a Danube River–floodplain system, Sieczko & Peduzzi (2014) recently concluded that for DOM, when residing in backwaters, the distinct conditions provide changed opportunities for processing of allochthonous carbon. In oceanic systems, vertical fluxes are important, in fluvial waters horizontal transport. The role of viruses in determining the proportion of organic material in horizontal transport *versus* processing and re‐mineralization in retention zones of fluvial waters remains understudied. Prokaryotic metabolic activity can apparently remove part of the continuous supply of terrestrial (allochthonous) DOC through the viral lysis of infected hosts. This involves promoting the uptake and respiration of material released from lysis by non‐infected cells. The magnitude of re‐mineralization through a bacteria–virus loop depends strongly on the number of passes through the loop, with increasingly more DOC respired at each turn (compare Pollard & Ducklow, 2011). Scaling up the related CO_2_ evasion to the system, catchment or even global level requires considering the (regionally variable) water surface area and a gas transfer velocity. A recent estimate of the global CO_2_ emission from streams and rivers predicts about 70% of the flux occurring over just 20% of the land surface (Raymond *et al*., 2013).

For a more reliable global up‐scaling, the virus‐related contribution remains to be investigated in more detail and in all kinds of fluvial systems. Anthropogenically altered stream and river morphology, e.g. by reducing wetlands and active floodplain areas in regulated rivers, may significantly impact that contribution, thus fostering horizontal downstream transport of solutes and particles (Schiemer, Hein & Peduzzi, 2006). Changes in habitat structure might also influence viral lifestyles and related processes.

Based on the information compiled herein, Table 3 lists a selection of neglected or understudied issues in the viral ecology of fluvial systems. In particular, greater attention should be given to targeted research on the horizontal transport of virus‐influenced solutes, particles, and the dissemination of viruses in flowing waters. This is of global relevance. The same holds true for improving our efforts in studying and scaling up the contribution of virus activity to global CO_2_ outgassing. Particular focus should be given to the effect of anthropogenic alterations to fluvial systems and climate change. The considerable knowledge gaps that remain should stimulate further research efforts to increase our understanding of flowing water systems.

**Table 3:** Choice and availability of key research for the understanding of fluvial virus ecology. Some issues have been studied previously, but all need a larger database from various climatic regions and from different types of fluvial waters

Research area	Partly available	Single or rare	Missing
Proportion of virus‐mediated host mortality *versus* grazing	X		
Proportion of viral lifestyles (lytic *versus* lysogenic)		X	
Diurnal studies			X
Impact of flooding, flow currents and turbulence on viruses			X
Role of suspended particles and biofilms on viral processes and survival		X	
Viral processes in sediments and at the aquatic–terrestrial interface		X	
Mechanisms of control on viral activity		X	
Viral diversity, viral effect on host diversity			X
Role of archaeal viruses			X
Role of eukaryotic viruses		X	
Metagenomic and proteomic studies			X
Chemical nature and bioreactivity of viral lysis products and lysozymes			X
Horizontal transport of virus‐influenced solutes and particles			X
Horizontal dissemination of virus particles			X
Contribution of virus activity to CO_2_ outgassing		X	
Biogeochemical relevance of the bacterial–viral loop		X	
Up‐scaling of virus‐related processes to the catchment scale and beyond			X

## IX. CONCLUSIONS

(1) One important driving factor in fluvial systems is their highly dynamic and heterogeneous nature, compared to the marine or lacustrine realm. Variable hydrology, edaphic heterogeneity and the broad range of trophic levels (even on narrow spatial scales such as in floodplains and backwaters) are essential elements of running waters, apparently impacting virus ecology significantly.

(2) Suspended particles, aggregates and biofilms are typical features of flowing waters; they probably influence virus‐related processes more strongly than in most lacustrine and oceanic systems. These structures can affect microbial (also viral) diversity, food‐web structure and biogeochemical cycles.

(3) Through repetitive viral lysis of infected bacterial hosts, the bacterial–viral loop appears to be significantly involved in removing part of the recalcitrant, terrestrial, dissolved organic matter from the water; increasingly more material is respired at each turn of the loop. A first appraisal of the viral contribution to CO_2_ outgassing from fluvial systems, synthesized from the scattered literature, reveals a potentially significant contribution on a global scale.

(4) Horizontal transport processes are relevant for virus dissemination and for virus‐influenced solutes and particles. Targeted research on such horizontal processes (to correspond with extensively conducted research on vertical transport in the oceans) is an important goal for the future.

(5) The significance of extensive anthropogenic alterations of fluvial systems, and of disturbance events by flooding, to viral lifestyles, host–virus diversity and virus activity needs to be investigated in detail, also in light of the effects of climate change.

(6) We need to develop comprehensive research programmes for a better understanding of virus‐related processes in flowing water systems. The outcome of such studies can be expected to contribute significantly to modern concepts of fluvial systems. Such concepts are necessary to understand and manage this important water resource better.

## References

[brv12202-bib-0001] Adriaenssens, E. M. & Cowan, D. A. (2014). Using signature genes as tools to assess environmental viral ecology and diversity. Applied and Environmental Microbiology 80, 4470–4480. 24837394 10.1128/AEM.00878-14 PMC4148782

[brv12202-bib-0002] Almeida, M. A. , Cunha, M. A. & Alcântara, F. (2001). Loss of estuarine bacteria by viral infection and predation in microcosm conditions. Microbial Ecology 42, 562–571. 12024239 10.1007/s00248-001-0020-1

[brv12202-bib-0003] Almeida, R. M. , Roland, F. , Cardoso, S. J. , Farjalla, V. F. , Bozelli, R. L. & Barros, N. O. (2015). Viruses and bacteria in floodplain lakes along a major Amazon tributary respond to distance to the Amazon River. Frontiers in Microbiology 6, 158 (doi: 10.3389/fmicb.2015.00158). 25788895 10.3389/fmicb.2015.00158 PMC4349158

[brv12202-bib-0004] Amado, A. M. , Cotner, J. B. , Cory, R. M. , Edhlund, B. L. & McNeill, K. (2015). Disentangling the interactions between photochemical and bacterial degradation of dissolved organic matter: amino acids play a central role. Microbial Ecology 69, 554–566. 25351141 10.1007/s00248-014-0512-4

[brv12202-bib-0005] Ankrah, N. Y. D. , May, A. L. , Middleton, J. L. , Jones, D. R. , Hadden, M. K. , Gooding, J. R. , LeCleir, G. R. , Wilhelm, S. W. , Campagna, S. R. & Buchan, A. (2014). Phage infection of an environmentally relevant marine bacterium alters host metabolism and lysate composition. The ISME Journal 8, 1089–1100. 24304672 10.1038/ismej.2013.216 PMC3996693

[brv12202-bib-0006] Auguet, J. C. , Montanie, H. , Delmas, D. , Hartmann, H. J. & Huet, V. (2005). Dynamic of virioplankton abundance and its environmental control in the Charente Estuary (France). Microbial Ecology 50, 337–349. 16328658 10.1007/s00248-005-0183-2

[brv12202-bib-0007] Azam, F. & Malfatti, F. (2007). Microbial structuring of marine ecosystems. Nature Reviews Microbiology 5, 782–791. 17853906 10.1038/nrmicro1747

[brv12202-bib-0008] Baker, P. W. & Leff, L. G. (2004). Seasonal patterns of abundance of viruses and bacteria in a Northeast Ohio (USA) stream. Archiv für Hydrobiologie 161, 225–233.

[brv12202-bib-0009] Barros, N. , Farjalla, V. F. , Soares, M. C. , Melo, R. C. N. & Roland, F. (2010). Virus‐bacterium coupling driven by both turbidity and hydrodynamics in an Amazonian floodplain lake. Applied and Environmental Microbiology 76, 7194–7201. 20833790 10.1128/AEM.01161-10 PMC2976232

[brv12202-bib-0010] Battin, T. J. , Kaplan, L. A. , Findlay, S. , Hopkinson, C. S. , Marti, E. , Packman, A. I. , Newbold, J. D. & Sabater, F. (2008). Biophysical controls on organic carbon fluxes in fluvial networks. Nature Geoscience 1, 95–100.

[brv12202-bib-0011] Battin, T. J. , Kaplan, L. A. , Findlay, S. , Hopkinson, C. S. , Marti, E. , Packman, A. I. , Newbold, J. D. & Sabater, F. (2009a). Corrigendum to: biophysical controls on organic carbon fluxes in fluvial networks. Nature Geoscience 2, 595.

[brv12202-bib-0012] Battin, T. J. , Luyssaert, S. , Kaplan, L. A. , Aufdenkampe, A. K. , Richter, A. & Tranvik, L. J. (2009b). The boundless carbon cycle. Nature Geoscience 2, 598–600.

[brv12202-bib-0013] Besemer, K. , Luef, B. , Preiner, S. , Eichberger, B. , Agis, M. & Peduzzi, P. (2009). Sources and composition of organic matter for bacterial growth in a large European river‐floodplain system (Danube, Austria). Organic Geochemistry 40, 321–331. 21151814 10.1016/j.orggeochem.2008.12.005 PMC2999831

[brv12202-bib-0014] Besemer, K. , Moeseneder, M. , Arrieta, J. M. , Herndl, G. J. & Peduzzi, P. (2005). Complexity of bacterial communities in a river‐floodplain system (Danube, Austria). Applied and Environmental Microbiology 71, 609–620. 15691909 10.1128/AEM.71.2.609-620.2005 PMC546682

[brv12202-bib-0015] Bettarel, Y. , Bouvier, T. , Agis, M. , Bouvier, C. , Chu, V. T. , Combe, M. , Mari, X. , Nghiem, M. N. , Nguyen, T. T. , Pham, T. T. , Pringault, O. , Rochelle‐Newall, E. , Torréton, J.‐P. & Tran, H. Q. (2011). Viral distribution and life strategies in the Bach Dang Estuary, Vietnam. Microbial Ecology 62, 143–154. 21390531 10.1007/s00248-011-9835-6

[brv12202-bib-0016] Bettarel, Y. , Bouvy, M. , Dumont, C. & Sime‐Ngando, T. (2006). Virus‐bacterium interactions in water and sediment of West African inland aquatic systems. Applied and Environmental Microbiology 72, 5274–5282. 16885276 10.1128/AEM.00863-06 PMC1538746

[brv12202-bib-0017] Breitbart, M. , Wegley, L. , Leeds, S. , Schoenfeld, T. & Rohwer, F. (2004). Phage community dynamics in hot springs. Applied and Environmental Microbiology 70, 1633–1640. 15006788 10.1128/AEM.70.3.1633-1640.2004 PMC368299

[brv12202-bib-0018] Brum, J. R. , Steward, G. F. , Jiang, S. C. & Jellison, R. (2005). Spatial and temporal variability of prokaryotes, viruses, and viral infections of prokaryotes in an alkaline, hypersaline lake. Aquatic Microbial Ecology 41, 247–260.

[brv12202-bib-0019] Brussaard, C. P. D. , Wilhelm, S. W. , Thingstad, F. , Weinbauer, M. G. , Bratbak, G. , Heldal, M. , Kimmance, S. A. , Middelboe, M. , Nagasaki, K. , Paul, J. H. , Schroeder, D. C. , Suttle, C. A. , Vaqué, D. & Wommack, K. E. (2008). Global‐scale processes with a nanoscale drive: the role of marine viruses. The ISME Journal 2, 575–578. 18385772 10.1038/ismej.2008.31

[brv12202-bib-0020] Danovaro, R. , Corinaldesi, C. , Dell'Anno, A. , Fuhrman, J. A. , Middelburg, J. J. , Noble, R. T. & Suttle, C. A. (2010). Marine viruses and global climate change. FEMS Microbiology Reviews 35, 993–1034. 10.1111/j.1574-6976.2010.00258.x 21204862

[brv12202-bib-0021] Danovaro, R. , Corinaldesi, C. , Filippini, M. , Fischer, U. R. , Gessner, M. O. , Jacquet, S. , Magagnini, M. & Velimirov, B. (2008a). Viriobenthos in freshwater and marine sediments: a review. Freshwater Biology 53, 1186–1213.

[brv12202-bib-0022] Danovaro, R. , Dell'Anno, A. , Corinaldesi, C. , Magagnini, M. , Noble, R. , Tamburini, C. & Weinbauer, M. (2008b). Major viral impact on the functioning of benthic deep‐sea ecosystems. Nature 454, 1084–1088. 18756250 10.1038/nature07268

[brv12202-bib-0023] Derx, J. , Farnleitner, A. H. , Zessner, M. & Blaschke, A. P. (2013). Evaluating the effect of temperature induced water viscosity and density fluctuations on virus and DOC removal during river bank filtration – a scenario analysis. River Systems 20, 169–184.

[brv12202-bib-0024] Downing, J. A. , Cole, J. J. , Duarte, C. M. , Middelburg, J. J. , Melack, J. M. , Prairie, Y. T. , Kortelainen, P. , Striegl, R. G. , McDowell, W. H. & Tranvik, L. J. (2012). Global abundance and size distribution of streams and rivers. Inland Waters 2, 229–236.

[brv12202-bib-0025] Farnell‐Jackson, E. A. & Ward, A. K. (2003). Seasonal patterns of viruses, bacteria and dissolved organic carbon in a riverine wetland. Freshwater Biology 48, 841–851.

[brv12202-bib-0026] Fischer, U. R. & Velimirov, B. (2002). High control of bacterial production by viruses in a eutrophic oxbow lake. Aquatic Microbial Ecology 27, 1–12.

[brv12202-bib-0027] Fischer, U. , Wieltschnig, C. , Kirschner, A. K. T. & Velimirov, B. (2003). Does virus‐induced lysis contribute significantly to bacterial mortality in the oxygenated sediment layer of shallow oxbow lakes? Applied and Environmental Microbiology 69, 5281–5289. 12957915 10.1128/AEM.69.9.5281-5289.2003 PMC194932

[brv12202-bib-0028] Fischer, U. R. , Wieltschnig, C. , Kirschner, A. K. T. & Velimirov, B. (2006). Contribution of virus‐induced lysis and protozoan grazing to benthic bacterial mortality estimated simultaneously in microcosms. Environmental Microbiology 8, 1394–1407. 16872403 10.1111/j.1462-2920.2006.01032.x

[brv12202-bib-0029] Flood, J. A. & Ashbolt, N. J. (2000). Virus‐sized particles are concentrated and maintained within wastewater wetland biofilms. Advances in Environmental Research 3, 403–411.

[brv12202-bib-0030] Fuhrman, J. A. & Schwalbach, M. (2003). Viral influence on aquatic bacterial communities. Biological Bulletin 204, 192–195. 12700152 10.2307/1543557

[brv12202-bib-0031] Goyal, S. M. , Gerba, C. P. & Britton, G. (1987). Phage Ecology. John Wiley & Sons, Inc., New York.

[brv12202-bib-0032] Haaber, J. & Middelboe, M. (2009). Viral lysis of Phaeocystis pouchetii: implications for algal population dynamics and heterotrophic C, N, and P cycling. The ISME Journal 3, 430–441. 19129863 10.1038/ismej.2008.125

[brv12202-bib-0033] Hennes, K. P. & Suttle, C. A. (1995). Direct counts of viruses in natural waters and laboratory cultures by epifluorescence microscopy. Limnology and Oceanography 40, 1050–1055.

[brv12202-bib-0034] Hewson, I. & Fuhrman, J. A. (2006). Viral impacts upon marine bacterioplankton assemblage structure. Journal of the Marine Biological Association of the United Kingdom 86, 577–589.

[brv12202-bib-0035] Hewson, I. , O'Neil, J. M. , Fuhrman, J. A. & Dennison, W. C. (2001). Virus‐like particle distribution and abundance in sediments and overlying waters along eutrophication gradients in two subtropical estuaries. Limnology and Oceanography 46, 1734–1746.

[brv12202-bib-0036] Jacquet, S. , Domaizon, I. , Chardon, C. & Personnic, S. (2013). Are small grazers and/or viruses a structuring factor of the free‐living bacterial community in Lake Geneva? Advances in Microbiology 3, 233–248.

[brv12202-bib-0037] Jacquet, S. , Miki, T. , Noble, R. , Peduzzi, P. & Wilhelm, S. (2010). Viruses in aquatic ecosystems: important advancements of the last 20 years and promising prospects in the field of microbial oceanography and limnology. Advances in Oceanography and Limnology 1, 71–101.

[brv12202-bib-0038] Jeanniard, A. , Dunigan, D. D. , Gurnon, J. R. , Agarkova, I. V. , Kang, M. , Vitek, J. , Duncan, G. , McClung, O. W. , Larsen, M. , Claverie, J. M. , Van Etten, J. L. & Blanc, G. (2013). Towards defining the chloroviruses: a genomic journey through a genus of large DNA viruses. BMC Genomics 14, 158–172. 23497343 10.1186/1471-2164-14-158 PMC3602175

[brv12202-bib-0039] Jiao, N. , Zhao, Y. , Luo, T. & Wang, X. (2006). Natural and anthropogenic forcing on the dynamics of virioplankton in the Yangtze river estuary. Journal of the Marine Biological Association of the UK 86, 543–550.

[brv12202-bib-0040] Junk, W. J. , Bayley, P. B. & Sparks, R. E. (1989). The flood pulse concept in river‐floodplain systems In Proceedings of the International Large River Symposium, Canadian Special Publication of Fisheries and Aquatic Sciences (Volume 165, ed. Dodge D. P.), pp. 110–127. Government of Canada Publications, Ottawa.

[brv12202-bib-0041] Kepner, R. L. , Wharton, R. A. & Suttle, C. A. (1998). Viruses in Antarctic lakes. Limnology and Oceanography 43, 1754–1761. 11543124 10.4319/lo.1998.43.7.1754

[brv12202-bib-0042] Kernegger, L. , Zweimüller, I. & Peduzzi, P. (2009). Effects of suspended matter quality and virus abundance on microbial parameters: experimental evidence from a large European river. Aquatic Microbial Ecology 57, 161–173. 24707113 10.3354/ame01341 PMC3972439

[brv12202-bib-0043] Lemke, M. , Wickstrom, C. & Leff, L. (1997). A preliminary study on the distribution of viruses and bacteria in lotic environments. Archiv für Hydrobiologie 141, 67–74.

[brv12202-bib-0044] Luef, B. , Aspetsberger, F. , Hein, T. , Huber, F. & Peduzzi, P. (2007). Impact of hydrology on free‐living and particle‐associated microorganisms in a river floodplain system (Danube, Austria). Freshwater Biology 52, 1043–1057.

[brv12202-bib-0045] Luef, B. , Neu, T. R. & Peduzzi, P. (2009a). Imaging and quantifying virus fluorescence signals on aquatic aggregates: a new method and its implication for aquatic microbial ecology. FEMS Microbiology Ecology 68, 372–380. 19416353 10.1111/j.1574-6941.2009.00675.x PMC2993471

[brv12202-bib-0046] Luef, B. , Neu, T. R. , Zweimüller, I. & Peduzzi, P. (2009b). Structure and composition of aggregates in two large European rivers, based on Confocal Laser Scanning Microscopy and image and statistical analysis. Applied and Environmental Microbiology 75, 5952–5962. 19633114 10.1128/AEM.00186-09 PMC2747869

[brv12202-bib-0047] Ma, L. , Sun, R. , Mao, G. , Yu, H. & Wang, Y. (2013). Seasonal and spatial variability of virioplankton abundance in Haihe River, China. BioMed Research International 2013, 1–10 (doi: 10.1155/2013/526362). 10.1155/2013/526362 PMC3703425 23844363

[brv12202-bib-0048] Madan, N. J. , Marshall, W. A. & Laybourn‐Parry, J. (2005). Virus and microbial loop dynamics over an annual cycle in three contrasting Antarctic lakes. Freshwater Biology 50, 1291–1300.

[brv12202-bib-0049] Mari, X. , Kerros, M. E. & Weinbauer, M. G. (2007). Virus attachment to transparent exopolymeric particles along trophic gradients in the southwest lagoon of New Caledonia. Applied and Environmental Microbiology 73, 5245–5252. 17586679 10.1128/AEM.00762-07 PMC1950989

[brv12202-bib-0050] Martínez Martínez, J. , Swan, B. K. & Wilson, W. H. (2014). Marine viruses, a genetic reservoir revealed by targeted viriomics. The ISME Journal 8, 1079–1088. 24304671 10.1038/ismej.2013.214 PMC3996692

[brv12202-bib-0051] Mathias, C. B. , Kirschner, A. K. T. & Velimirov, B. (1995). Seasonal variations of virus abundance and viral control of bacterial production in a backwater system of the Danube River. Applied and Environmental Microbiology 61, 3734–3740. 16535153 10.1128/aem.61.10.3734-3740.1995 PMC1388715

[brv12202-bib-0052] Maurice, C. F. , Bouvier, C. , de Wit, R. & Bouvier, T. (2013). Linking the lytic and lysogenic bacteriophage cycles to environmental conditions, host physiology and their variability in coastal lagoons. Environmental Microbiology 15, 2463–2475. 23581698 10.1111/1462-2920.12120

[brv12202-bib-0053] Mei, M. L. & Danovaro, R. (2004). Virus production and life strategies in aquatic sediments. Limnology and Oceanography 49, 459–470.

[brv12202-bib-0054] Mojica, K. D. A. & Brussaard, C. P. D. (2014). Factors affecting virus dynamics and microbial host‐virus interactions in marine environments. FEMS Microbiology Ecology 89, 495–515. 24754794 10.1111/1574-6941.12343

[brv12202-bib-0055] Newbold, J. D. (1992). Cycles and spirals of nutrients In The Rivers Handbook (Volume I, eds Calow P. and Petts G. E.), pp. 379–408. Blackwell Scientific, Oxford.

[brv12202-bib-0056] Nilsson, C. , Reidy, C. A. , Dynesius, M. & Revenga, C. (2005). Fragmentation and flow regulation of the world's large river systems. Science 308, 405–408. 15831757 10.1126/science.1107887

[brv12202-bib-0057] Ortmann, A. C. , Metzger, R. C. , Liefer, J. D. & Novoveska, L. (2011). Grazing and viral lysis vary for different components of the microbial community across an estuarine gradient. Aquatic Microbial Ecology 65, 143–157.

[brv12202-bib-0058] Parada, V. , Herndl, G. J. & Weinbauer, M. G. (2006). Viral burst size of heterotrophic prokaryotes in aquatic systems. Journal of the Marine Biological Association of the United Kingdom 86, 613–621.

[brv12202-bib-0059] Payet, J. P. & Suttle, C. A. (2013). To kill or not to kill: the balance between lytic and lysogenic viral infection is driven by trophic status. Limnology and Oceanography 58, 465–474.

[brv12202-bib-0060] Peduzzi, P. (2013). Ecological aspects of viruses in a large river (Danube, Austria): a widely ignored field of inland water ecology. Danube News 27, 11–14.

[brv12202-bib-0061] Peduzzi, P. (2015). Aquatic viruses and global climate change In Climate Change and Microbial Ecology: Current Research and Future Trends (ed. Marxsen J.). Caister Academic Press, Norfolk, in press.

[brv12202-bib-0062] Peduzzi, P. , Agis, M. & Luef, B. (2013). Evaluation of confocal laser scanning microscopy for enumeration of virus‐like particles in aquatic systems. Environmental Monitoring and Assessment 185, 5411–5418. 23108709 10.1007/s10661-012-2955-8 PMC4970652

[brv12202-bib-0063] Peduzzi, P. , Aspetsberger, F. , Hein, T. , Huber, F. , Kargl‐Wagner, S. , Luef, B. & Tachkova, Y. (2008). Dissolved organic matter (DOM) and bacterial growth in floodplains of the River Danube under varying hydrological connectivity. Fundamental and Applied Limnology 171, 49–61.

[brv12202-bib-0064] Peduzzi, P. , Gruber, M. , Gruber, M. & Schagerl, M. (2014). The virus's tooth – cyanophages affect an African flamingo population in a bottom up cascade. The ISME Journal 8, 1346–1351. 24430484 10.1038/ismej.2013.241 PMC4030235

[brv12202-bib-0065] Peduzzi, P. & Luef, B. (2008). Viruses, bacteria and suspended particles in a backwater and main channel site of the Danube (Austria). Aquatic Sciences 70, 186–194. 21151810 10.1007/s00027-008-8068-3 PMC2999825

[brv12202-bib-0066] Peduzzi, P. & Luef, B. (2009). Viruses In Encyclopedia of Inland Waters (ed. Likens G. E.), pp. 279–294. Elsevier, Oxford.

[brv12202-bib-0067] Peduzzi, P. & Schiemer, F. (2004). Bacteria and viruses in the water column of tropical freshwater reservoirs. Environmental Microbiology 6, 707–715. 15186349 10.1111/j.1462-2920.2004.00602.x

[brv12202-bib-0068] Peduzzi, P. & Weinbauer, M. G. (1993). Effect of concentrating the virus‐rich 2–200 nm size fraction of seawater on the formation of algal flocs (marine snow). Limnology and Oceanography 38, 1562–1565.

[brv12202-bib-0069] Pinto, F. , Larsen, S. & Casper, P. (2013). Viriobenthos in aquatic sediments: variability in abundance and production and impact on the C‐cycle. Aquatic Sciences 75, 571–579.

[brv12202-bib-0070] Pollard, P. C. & Ducklow, H. (2011). Ultrahigh bacterial production in a eutrophic subtropical Australian river: does viral lysis short‐circuit the microbial loop? Limnology and Oceanography 56, 1115–1129.

[brv12202-bib-0071] Pradeep Ram, A. S. , Palesse, S. , Colombet, J. , Thouvenot, A. & Sime‐Ngando, T. (2014). The relative importance of viral lysis and nanoflagellate grazing for prokaryote mortality in temperate lakes. Freshwater Biology 59, 300–311.

[brv12202-bib-0072] Pradeep Ram, A. S. & Sime‐Ngando, T. (2014). Distinctive patterns in prokaryotic community composition in response to viral lysis and flagellate grazing in freshwater microcosms. Freshwater Biology 59, 1945–1955.

[brv12202-bib-0073] Prangishvili, D. , Forterre, P. & Garrett, R. A. (2006). Viruses of the Archaea: a unifying view. Nature Reviews 4, 837–848. 10.1038/nrmicro1527 17041631

[brv12202-bib-0074] Proctor, L. M. & Fuhrman, J. A. (1990). Viral mortality of marine bacteria and cyanobacteria. Nature 343, 60–62.

[brv12202-bib-0075] Raymond, P. A. , Hartmann, J. , Lauerwald, R. , Sobek, S. , McDonald, C. , Hoover, M. , Butman, D. , Striegl, R. , Mayorga, E. , Humborg, C. , Kortelainen, P. , Dürr, H. , Meybeck, M. , Ciais, P. & Guth, P. (2013). Global carbon dioxide emission from inland waters. Nature 503, 355–359. 24256802 10.1038/nature12760

[brv12202-bib-0076] Sandaa, R.‐A. , Gómez‐Consarnau, L. , Pinhassi, J. , Riemann, L. , Malits, A. , Weinbauer, M. G. , Gasol, J. M. & Thingstad, T. F. (2009). Viral control of bacterial biodiversity – evidence from a nutrient‐enriched marine mesocosm experiment. Environmental Microbiology 11, 2585–2597. 19558511 10.1111/j.1462-2920.2009.01983.x

[brv12202-bib-0077] Schiemer, F. , Hein, T. & Peduzzi, P. (2006). Hydrological control of system characteristics of floodplain lakes. Ecohydrology and Hydrobiology 6, 7–18.

[brv12202-bib-0078] Seymour, J. R. , Seuront, L. , Doubell, M. , Waters, R. L. & Mitchell, J. G. (2006). Microscale patchiness of virioplankton. Journal of the Marine Biological Association of the UK 86, 551–561.

[brv12202-bib-0079] Sheik, A. R. , Brussaard, C. P. D. , Lavik, G. , Lam, P. , Musat, N. & Krupke, A. (2014). Responses of the coastal bacterial community to viral infection of the algae *Phaeocystis globosa* . The ISME Journal 8, 212–225. 23949664 10.1038/ismej.2013.135 PMC3869014

[brv12202-bib-0080] Shelford, E. J. , Jorgensen, N. O. G. , Rasmussen, S. , Suttle, C. A. & Middelboe, M. (2014). Dissecting the role of viruses in marine nutrient cycling: bacterial uptake of D‐ and L‐amino acids released by viral lysis. Aquatic Microbial Ecology 73, 235–243.

[brv12202-bib-0081] Shelford, E. J. , Middelboe, M. , Moller, E. F. & Suttle, C. A. (2012). Virus‐driven nitrogen cycling enhances phytoplankton growth. Aquatic Microbial Ecology 66, 41–46.

[brv12202-bib-0082] Sieczko, A. , Maschek, M. & Peduzzi, P. (2015). Algal extracellular release in river‐floodplain dissolved organic matter: response of extracellular enzymatic activity during a post‐flood period. Frontiers in Microbiology 6, 80 (doi: 10.3389/fmicb.2015.00080). 25741326 10.3389/fmicb.2015.00080 PMC4330910

[brv12202-bib-0083] Sieczko, A. & Peduzzi, P. (2014). Origin, enzymatic response and fate of dissolved organic matter during flood and non‐flood conditions in a river‐floodplain system of the Danube (Austria). Aquatic Sciences 76, 115–129. 24415892 10.1007/s00027-013-0318-3 PMC3883529

[brv12202-bib-0084] Simek, K. , Pernthaler, J. , Weinbauer, M. G. , Hornak, K. , Dolan, J. R. , Nedoma, J. , Masin, M. & Amann, R. (2001). Changes in bacterial community composition and dynamics and viral mortality rates associated with enhanced flagellate grazing in a mesoeutrophic reservoir. Applied and Environmental Microbiology 67, 2723–2733. 11375187 10.1128/AEM.67.6.2723-2733.2001 PMC92931

[brv12202-bib-0085] Slováckova, H. & Marsálek, B. (2008). Virioplankton and microbial communities in two Czech rivers (Svratka and Morava River). Aquatic Sciences 70, 282–291.

[brv12202-bib-0086] Stocker, R. (2012). Marine microbes see a sea of gradients. Science Reviews 338, 628–633. 10.1126/science.1208929 23118182

[brv12202-bib-0087] Suttle, C. A. (2007). Marine viruses – major players in the global ecosystem. Nature Reviews Microbiology 5, 801–812. 17853907 10.1038/nrmicro1750

[brv12202-bib-0088] Thingstad, T. F. (2000). Elements of a theory for the mechanisms controlling abundance, diversity, and biogeochemical role of lytic bacterial viruses in aquatic systems. Limnology and Oceanography 45, 1320–1328.

[brv12202-bib-0089] Thingstad, T. F. & Lignell, R. (1997). Theoretical models for the control of bacterial growth rate, abundance, diversity and carbon demand. Aquatic Microbial Ecology 13, 19–27.

[brv12202-bib-0090] Thomas, R. , Berdjeb, L. , Sime‐Ngando, T. & Jacquet, S. (2011). Viral abundance, production, decay rates and life strategies (lysogeny versus lysis) in Lake Bourget (France). Environmental Microbiology 13, 616–630. 21054737 10.1111/j.1462-2920.2010.02364.x

[brv12202-bib-0091] Thurber, R. V. , Haynes, M. , Breitbart, M. , Wegley, L. & Rohwer, F. (2009). Laboratory procedures to generate viral metagenomes. Nature Protocols 4, 470–483. 19300441 10.1038/nprot.2009.10

[brv12202-bib-0092] Tvarogova, J. (2010). Viral abundance, lytic life cycles and lysogeny in a riverine environment (Danube, Austria). MSc Thesis: University of Vienna, Vienna.

[brv12202-bib-0093] Van Etten, J. L. , Lane, L. C. & Meints, R. H. (1991). Viruses and viruslike particles of eukaryotic algae. Microbiological Reviews 55, 586–620. 1779928 10.1128/mr.55.4.586-620.1991 PMC372839

[brv12202-bib-0094] Weinbauer, M. G. (2004). Ecology of prokaryotic viruses. FEMS Microbiology Reviews 28, 127–181. 15109783 10.1016/j.femsre.2003.08.001

[brv12202-bib-0095] Weinbauer, M. G. , Bettarel, Y. , Cattaneo, R. , Luef, B. , Maier, C. , Motegi, C. , Peduzzi, P. & Mari, X. (2009). Viral ecology of organic and inorganic particles in aquatic systems: avenues for further research. Aquatic Microbial Ecology 57, 321–341. 27478304 10.3354/ame01363 PMC4962909

[brv12202-bib-0096] Weinbauer, M. G. , Nedoma, J. , Christaki, U. & Simek, K. (2003). Comparing the effects of resource enrichment and grazing on viral production in a meso‐eutrophic reservoir. Aquatic Microbial Ecology 31, 137–144.

[brv12202-bib-0097] Weinbauer, M. G. & Peduzzi, P. (1995). Effect of virus‐rich high molecular weight concentrates of seawater on the dynamics of dissolved amino acids and carbohydrates. Marine Ecology Progress Series 127, 245–253.

[brv12202-bib-0098] Weitz, J. S. , Stock, C. A. , Wilhelm, S. W. , Bourouiba, L. , Coleman, M. L. , Buchan, A. , Fellows, M. J. , Fuhrman, J. A. , Jover, L. F. , Lennon, J. T. , Middelboe, M. , Sonderegger, D. L. , Suttle, C. A. , Taylor, B. P. , Thingstad, T. F. , *et al* (2014). A multitrophic model to quantify the effects of marine viruses on microbial food webs and ecosystem processes. The ISME Journal 9, 1352–1364 (doi: 10.1038/ismej.2014.220). 10.1038/ismej.2014.220 PMC4438322 25635642

[brv12202-bib-0099] Weitz, J. S. & Wilhelm, S. W. (2012). Ocean viruses and their effects on microbial communities and biogeochemical cycles. F1000 Biology Reports 4, 1–17. Available at http://f1000.com/reports/b/4/17. Accessed 5.9.2012. 22991582 10.3410/B4-17 PMC3434959

[brv12202-bib-0100] Wilhelm, S. W. & Matteson, A. R. (2008). Freshwater and marine virioplankton: a brief overview of commonalities and differences. Freshwater Biology 53, 1076–1089.

[brv12202-bib-0101] Wilhelm, S. W. & Suttle, C. A. (1999). Viruses and nutrient cycles in the sea. BioScience 49, 781–788.

[brv12202-bib-0102] Williamson, K. E. , Harris, J. V. , Green, J. C. , Rahman, F. & Chambers, R. M. (2014). Stormwater runoff drives viral community composition changes in inland freshwaters. Frontiers in Microbiology 5 (doi: 10.3389/fmicb.2014.00105). 10.3389/fmicb.2014.00105 PMC3954104 24672520

[brv12202-bib-0103] Wommack, K. E. & Colwell, R. R. (2000). Virioplankton: viruses in aquatic ecosystems. Microbiology and Molecular Biology Reviews 64, 69–114. 10704475 10.1128/mmbr.64.1.69-114.2000 PMC98987

[brv12202-bib-0104] Yamada, T. , Shimomae, A. , Furukawa, S. & Takehara, J. (1993). Widespread distribution of *Chlorella* viruses in Japan. Bioscience, Biotechnology, and Biochemistry 57, 733–739. 10.1271/bbb.57.733 7763775

[brv12202-bib-0105] Yang, Y. & Griffiths, M. W. (2013). Comparative persistence of subgroups of F‐specific RNA phages in river water. Applied and Environmental Microbiology 79, 4564–4567. 23686274 10.1128/AEM.00612-13 PMC3719516

[brv12202-bib-0106] Yolken, R. H. , Jones‐Brando, L. , Dunigan, D. D. , Kannan, G. , Dickerson, F. , Severance, E. , Sabunciyan, S. , Talbot, C. C. Jr. , Prandovszky, E. , Gurnon, J. R. , Agarkova, I. V. , Leister, F. , Gressitt, K. L. , Chen, O. , Deuber, B. , *et al* (2014). Chlorovirus ATCV‐1 is part of the human oropharyngeal virome and is associated with changes in cognitive functions in humans and mice. Proceedings of the National Academy of Sciences of the United States of America 111, 16106–16111. 25349393 10.1073/pnas.1418895111 PMC4234575

[brv12202-bib-0107] Zeigler Allen, L. , Ishoey, T. , Novotny, M. A. , McLean, J. S. , Lasken, R. S. & Williamson, S. J. (2011). Single virus genomics: a new tool for virus discovery. PLoS One 6, e17722 (doi: 10.1371/journal.pone.0017722). 21436882 10.1371/journal.pone.0017722 PMC3059205

[brv12202-bib-0108] Zweimüller, I. , Zessner, M. & Hein, T. (2008). Effects of climate change on nitrate loads in a large river: the Austrian Danube as example. Hydrological Processes 22, 1022–1036.

